# Comparing and optimizing ultraviolet germicidal irradiation systems use for patient room terminal disinfection: an exploratory study using radiometry and commercial test cards

**DOI:** 10.1186/s13756-018-0317-1

**Published:** 2018-02-22

**Authors:** Vincent Masse, Michael J. Hartley, Michael B. Edmond, Daniel J. Diekema

**Affiliations:** 1 Department of Internal Medicine, University of Iowa Carver College of Medicine and University of Iowa Hospitals and Clinics, 200 Hawkins Drive # C512-GH, Iowa City, IA 52242 USA; 2 Department of Microbiology and Infectious Diseases, Faculty of Medicine and Health Sciences, University of Sherbrooke, Sherbrooke, Québec Canada; 3 Department of Hospital Administration, University of Iowa Hospitals and Clinics, Iowa City, IA USA; 4 Office of Clinical Quality Safety and Performance Improvement, University of Iowa Hospitals and Clinics, Iowa City, IA USA; 5 Department of Pathology, University of Iowa Carver College of Medicine and University of Iowa Hospitals and Clinics, Iowa City, IA USA

**Keywords:** UVGI, Ultraviolet, Terminal room disinfection, Radiometry, Test cards

## Abstract

**Background:**

Ultraviolet germicidal irradiation (UVGI) systems are gaining popularity, however objective comparisons of their characteristics are lacking. While environmental cultures and reduction of hospital-associated infections rates are excellent study endpoints, they are impractical for centers with limited resources who want to compare or optimize UVGI systems use.

**Methods:**

We evaluated radiometry and commercial test cards, two simple and low cost tools, to compare 2 full size UVGI systems (Tru-D and Optimum-UV Enlight) and 2 small units (Lumalier EDU 435 and MRSA-UV Turbo-UV).

**Results:**

Radiometry-derived output curves show that if both large devices emit enough energy to reach *C. difficile* lethal doses at 10 ft, the reduction in output in distance is almost perfectly logarithmic. In a patient room environment, Enlight and Tru-D performed similarly when compared using radiometry and commercial test cards. The two small devices reached *C. difficile* range around the bathroom with the device raised above the floor, but longer times are needed.

**Conclusions:**

Despite different workflows and price points, no clear superiority emerges between Tru-D and Enlight. Bathroom disinfection should be dealt with separately from the main room and small, cheaper units can be used. Radiometry and commercial test cards are promising ways to compare UVGI systems, but further validation is needed using correlation with environmental cultures.

**Trial registration:**

Not applicable.

## Background

Ultraviolet germicidal irradiation (UVGI) systems for patient room terminal disinfection are gaining popularity. Surface contamination is linked to transmission of multi-drug resistant pathogens [1, 2], and maintaining consistent excellence in manual disinfection can be difficult [3].

No-touch systems using UVC light have been shown to reduce room contamination of both vegetative bacteria and bacterial spores in multiple environmental studies [4]. Their effectiveness at reducing HAIs has also been studied, but results are somewhat conflicting [5]. Recently, a multicenter, randomized study [6] showed a 30% reduction in transmission events for methicillin-resistant *Staphylococcus aureus* (MRSA), vancomycin-resistant *Enterococcus* (VRE) and *Clostridium difficile* using UVGI as an adjunct to standard cleaning. Although impressive, this translates to a “number needed to treat” of 575 exposure-days. In practice, this means disinfecting 115 rooms (assuming a 5-day average stay) to prevent 1 transmission event.

The UVGI market is currently poorly regulated, and there are no agreed-upon standards to evaluate devices [7]. As expected, different test parameters influence the results obtained when comparing systems [8]. Moreover, UVGI systems vary greatly in their complexity (some have sophisticated sensor systems while others are basically lamps on a timer switch). Workflows also differ, with some devices requiring multiple short placements and others a single, longer cycle to process the same room.

Selecting the optimal device for use in a specific institution can be difficult since there are surprisingly few head to head comparisons of devices or published accounts of the selection process [9–11]. Performing local prospective evaluations of UVGI systems is burdensome and requires substantial resources; environmental cultures are complex and labor-intensive, and evaluating the impact on HAIs requires prolonged observations that are impractical. Then, once UVGI is implemented, how can a hospital conveniently and rapidly determine if the process is optimal for the environment it is used in (is device and placement in the room optimal to limit shadowing, are cycles long enough to ensure satisfactory UVC dose, etc.)?

We explored two simple, low-cost tools to objectively compare UVGI devices and assess their energy dispersion in patient rooms: UVC radiometry and commercial test cards.

## Methods

### Setting and tested devices

The University of Iowa Hospitals and Clinics is a 761-bed tertiary and quaternary care teaching hospital in Iowa City, Iowa. The hospital spans multiple adjacent pavilions, including a state of the art recently opened children’s hospital. Census is chronically high, and room turnover is of paramount importance.

UVGI using the Tru-D system (Tru-D Smart UVC, Memphis, TN) has been used as an adjunct to manual cleaning for contact isolation patient rooms since 2014. The devices are always run on the “Spore Cycle” setting, leading to prolonged disinfection times, often more than 60 min, depending on room size, window size, furniture position and device placement. In many areas, the room and the bathroom are irradiated at the same time, using a single Tru-D machine. In other parts of the hospital, the bathrooms get their dedicated cycles using a smaller unit (EDU 435, Lumalier Corporation, Memphis, TN).

The Clorox Company (Oakland, CA) provided us with a Clorox Healthcare Optimum-UV Enlight device and a box of their Dose Verify™ test cards for our experiments. Dose Verify™ cards are colorimetric cards that change color from yellow to green when exposed to UVC light. A color scale directly on the card allows for comparison with MRSA and *C. difficile* lethal energy ranges. The cards are calibrated for use with Clorox Healthcare products.

We chose to compare our EDU 435 with another small device, the Turbo-UV (MRSA-UV, Palm Beach, FL).

None of the manufacturers provided any input into the study or in the drafting of the manuscript.

Table 1 compares the 4 devices we tested.

**Table 1:** UVGI devices comparison

	Large units for patient room disinfection		Small units for bathrooms and other confined spaces	
Name (manufacturer)	Tru-D (Tru-D SmartUVC, Memphis, TN)	Enlight (Clorox Healthcare - Optimum UV, Oakland, CA)	EDU 435 (Lumalier, Memphis, TN)	Turbo-UV (MRSA-UV, Palm Springs, FL)
Workflow and control	• One room, one device placement • On-board sensor system calculates cycle time based on reflected UVC dose • “Bacteria” and “Spore” cycles • Cycle time can vary greatly • Remote control • Cloud-based use data log	• One room, multiple device placement • Cycle time, number of placements and positions determined by user • Test cards available to help with the process • Sensor system used to detect intrusion in the room while device in use • Control panel on device • App-based remote • Cloud-based use data log	• Delay start function • Timer	• Remote control • Timer
Available literature	Most studied UVGI system	A few available studies	No studies available	No studies available
Price point (approximate, USD)	$90,000	$45,000	$4000	$600

### Power and energy curves

We used a portable compact radiometer with data “auto logging” capabilities optimized for the 254 nm band (General UV254SD, General Tools, New York, NY) to measure the power output of the UVGI devices every second over a continuous period of 5 min at a distance of 5, 8, 10 and 15 ft (1.5, 2.4, 3 and 4.6 m, respectively) for the large units and 5 and 8 ft (1.5 and 2.4 m, respectively) for the small units. Each device was tested separately in an unoccupied but furnished patient room with the sensor aimed directly at the device, 4 ft (1.2 m) above the ground for Tru-D and Enlight and 6 in. (15.2 cm) for Turbo-UV and EDU-435. A single official 5-min reading was performed with each device, due to time constraints.

Mapping the power vs. time curves allowed us to calculate the area under the curve (using a Microsoft Excel algorithm based on the Riemann summation) to estimate the cumulative UVC energy (or UVC dose) emitted by the devices over the 5 min of the experiment. These values can then be compared to pathogen-specific lethal doses available in the literature (references [12, 13] for MRSA and *C. difficile* spores).

### Live tests

We compared the Tru-D and Enlight standard operating protocols in a large empty patient room (22 X 24 ft or 6.7 X 7.3 m). Tru-D was placed in the center of the room and run on a “Spore Cycle” for 60 min according to its internal sensors and software. Enlight was used for 5 min in 3 different strategic areas around the room, as recommended by the manufacturer. In both cases, the bathroom door was left open to allow for its simultaneous disinfection. The furniture in the room was not moved, to create a “worst-case” scenario for shadowing.

We selected 10 areas of clinical interest using our infection prevention experience and the input of a certified infection preventionist (see Table 2) where we placed a Dose Verify™ card for the duration of the disinfection process. Color changes were then graded as “MRSA range” or “*C. difficile range*” using the color scale by the visual assessment of two of the authors (VM and MJH). We obtained UVC lethal dose information from the available literature [12, 13].

**Table 2:** Live tests results with the large units

Area	Power reading (mW/cm^2^) Tru-D Center 1 h runtime	Energy estimation (mJ) Tru-D Center 1 h runtime	*Dose Verify ™* Result	Sum of Power readings (mW/cm^2^) Enlight 3 spots X 5 min	Energy estimation (mJ) Enlight 3 spots X 5 min	*Dose Verify ™* Result
BR faucet	0	0	No change	0.021	6.30	No change
Flush handle	0	0	MRSA range	0.007	2.10	No change
HH station	0.257	925	*C. diff* range	0.829	249	*C. diff* range
Keyboard	0.0628	226	*C. diff* range	0.9654	289	*C. diff* range
Heart monitor	0.319	1148	*C. diff* range	0.1875	56	*C. diff* range
Sphygmo dial	0.350	1260	*C. diff* range	0.336	101	*C. diff* range
Crib	0.243	875	*C. diff* range	0.3125	94	*C. diff* range
Under table	0	0	MRSA range	0.429	129	*C. diff* range
IV pump	0.684	2462	*C. diff* range	0.614	184	*C. diff* range
Floor, corner	0.063	227	MRSA range	0.061	18	No change
% of areas in range	No change	30%	10%	No change	20%	30%
	MRSA or *C. diff*	70%	90%	MRSA or *C. diff*	80%	70%
	MRSA alone	*0%*	30%	MRSA alone	10%	0%
	*C. diff*	70%	60%	*C. diff*	70%	70%

*BR* bathroom, *HH* hand hygiene, *Sphygmo* sphygmomanometer, *C. diff C. difficile*. The MRSA lethal energy dose is 12 mJ. The *C. difficile* lethal energy dose is 38.5 mJ

In a second experiment, we performed live radiometry readings. One of us (MJH) wore radiation opaque protective equipment and performed measurements with the UVGI device in use at the same areas after a 5-min warm-up period. Assuming a constant and stable power output over a cycle, we crudely estimated the cumulative energy output in one area by multiplying the reading by the cycle duration. For Enlight, measurements obtained with the devices in the 3 placements were multiplied by 5 min each and then added.

To test the small units, we placed each device in the adjacent bathroom (9.25 X 6.25 ft or 2.8 X 1.9 m), with the door closed. We did not perform live readings, but we used the test cards in strategic areas (see Table 3). Seeing the results of our first test with the Turbo device, we modified the protocol by raising the UVGI device 20 in. (51 cm) above the floor (we put the device on top of the – empty – bathroom trashcan flipped upside down to perform the remaining tests). The cycle time was 15 min for each device.

**Table 3:** Live tests results with the small units

Area		Turbo-UV on the floor	Turbo-UV raised 20 in.	EDU 435 raised 20 in.
Bathroom faucet		No change	*C. difficile* range	*C. difficile* range
Toilet flush handle		*C. difficile* range	*C. difficile* range	*C. difficile* range
Toilet seat		No change	*C. difficile* range	*C. difficile* range
Shower drain		*C. difficile* range	*C. difficile* range	*C. difficile* range
Shower back wall		*C. difficile* range	*C. difficile* range	*C. difficile* range
Door handle		*C. difficile* range	*C. difficile* range	*C. difficile* range
Toilet paper roll		*C. difficile* range	*C. difficile* range	*C. difficile* range
% of areas in range	No change	29%	0%	0%
	MRSA or *C. difficile*	71%	100%	100%
	MRSA alone	0%	0%	0%
	*C. difficile*	71%	100%	100%

## Results

### Power and energy curves

**Fig. 1:**
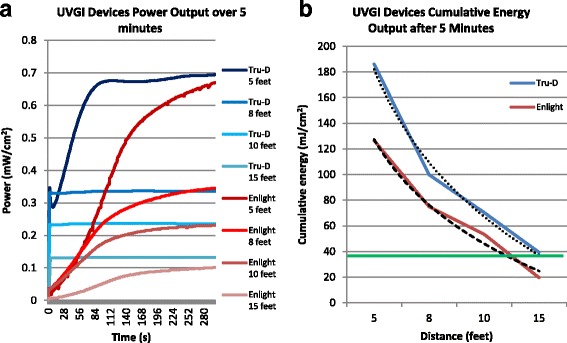
Power and Energy Output of the Large UVGI Units. **a** The power output in time is expressed in mW/cm^2^ for each distance. **b** The curves show the cumulative energy (in mJ/cm^2^) at each distance for each device. A power of 1 watt (W) applied for 1 second (s) produces 1 joule (J) of energy. The green line represents the *C. difficile* lethal energy dose (38.5 mJ). The dashed curves are the logarithmic regressions. 5 feet = 1.5 m; 8 feet = 2.4 m; 10 feet = 3 m; 15 feet = 4.6 m

Figure 1 displays the power and energy curves obtained with the large units. The Tru-D device is more powerful at every distance, but the difference decreases with increasing distance. Both devices are powerful enough to kill *C. difficile* spores at 5, 8 and 10 ft over 5 min based on the cumulative energy received (Tru-D: 186.2, 99,1 and 70.4 mJ/cm^2^ respectively; Enlight: 126.6, 75.0 and 53.4 mJ/cm^2^ respectively) and the available lethal UVC dose (38.5 mJ). Tru-D emitted lethal energy for *C. difficile* at 15 ft, contrarily to Enlight (39.4 vs. 19.6 mJ/cm^2^). Tru-D’s output plateaus more rapidly than Enlight. The reduction in power over distance is almost perfectly logarithmic (see the dashed regression curves on Fig. 1b).

**Fig. 2:**
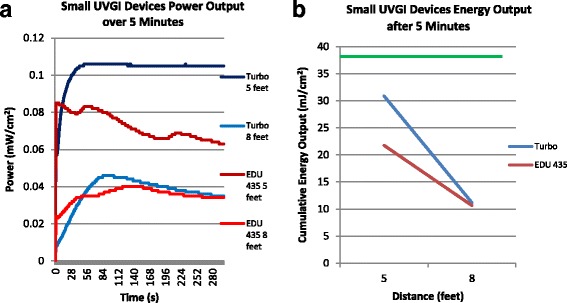
Power and Energy Output of the Small UVGI Units. **a** The power output in time is expressed in mW/cm^2^ for each distance. **b** The curves show the cumulative energy (in mJ/cm^2^) at each distance for each device. A power of 1 watt (W) applied for 1 second (s) produces 1 joule (J) of energy. The green line represents the *C. difficile* lethal energy dose (38.5 mJ). 5 feet = 1.5 m; 8 feet = 2.4 m

Figure 2 shows the same curves, but for the small devices. The Turbo-UV device is more powerful than the EDU 435 device, but none of the small devices reached *C. difficile* range in 5 min (Turbo UV: 30.9 and 11.2 mJ/cm^2^ respectively; EDU-435: 21.8 and 10.6 mJ/cm^2^ respectively).

### Live tests

Table 2 presents the results of the live tests for the large devices. Radiometry estimates slightly favor the Enlight device for both MRSA and *C. difficile* ranges combined (80% areas within range vs. 70%), whereas test card results favor Tru-D (90% vs. 70%). For *C. difficile*-range disinfection, radiometry results were similar for both systems (70%), while test cards show a small advantage with the Enlight system (70% vs. 60%).

Radiometry estimates correlate reasonably well with the test cards. If 20% of data points did not match (4/20), in half the cases the radiometry reading was 0, suggesting that we simply did not catch the right angle with the radiometer. In the last 2 cases, radiometry picked up more energy than did the test cards.

The results also show very poor energy diffusion in the bathroom using a device in the main room alone.

However, as shown with Table 3, both small devices in the bathroom perform very well. With the device on the floor, we realized that we did not have a direct line of sight with the toilet seat or with the faucet, so no energy could reach that area. Raising the device using what was available in the room (i.e. the trash can) solved the problem.

## Discussion

We explored avenues other than environmental cultures to evaluate, compare and optimise use of UVGI devices. Our energy and power curves demonstrate well that the power (and hence the energy delivered) by UVGI devices falls rapidly with distance from the unit. However, these same curves show that both the Tru-D and Enlight devices emit more than enough energy in 5 min to effectively kill *C. difficile* spores at 10 ft (3 m). Most surfaces in most post patient rooms will be within a 10-ft (3 m) radius of a strategically placed UVGI device.

Tru-D has automated sensors that measure the reflected UVC dose back to the device. The manufacturer claims, with published data in support [13], that the high power of the device combined with the sensors allows for “total room disinfection” [14], and that even shadowed areas can be disinfected due to the reflection of UVCs on walls and surfaces. The cost of this is longer cycle times, especially in large rooms.

Looking at our live tests, we did measure significantly more energy (both with radiometry and using test cards) in the shadowed far corner of the room with Tru-D than with Enlight, albeit not to *C. difficile*-disinfection dose. However, another dark area, the underside of the bedside rolling table, saw better results with the Enlight device, presumably because its taller design allowed for a better line of sight under the table. Overall, the Tru-D system did not perform significantly better in our tests than the Enlight device.

While using sensors can be reassuring and limit inter-user variability, our results suggest that shorter times with more placements around the room provide essentially comparable results, using a fraction of the total time (and for a fraction of the cost). The impact on room turnaround time is worth considering.

Our data show that bathrooms (or other areas adjoining the room – anterooms, large closets, etc.) should be dealt with separately from the main room. Using a small device in the bathroom (ideally raised above the floor) simultaneously to the main room cycle (using 2 or 3 5-min placements in the main room) allows the process to be complete in less than 20 min and the room to be ready for its next occupant. These small devices could possibly have other applications in healthcare, notably for ambulances [15].

Shadowing will always remain a limitation of UVGI. While reflection on surfaces can circumvent part of this problem, it is unrealistic to believe that ALL surfaces can be reached while using a practical methodology. Attention should be focused on relevant areas in the patient environment, notably “high touch” surfaces, and ensuring that they are in line of sight of the device when it is used. UVGI operators should be adequately trained to achieve this. Large UVGI manufacturers such as Clorox and Tru-D SmartUVC offer training to their customers.

A portable radiometer is inexpensive (USD $300–800) and easy to use, but few articles using this tool have been published [15–17]. Recently, Lindsley et al. [15] used environmental cultures of *Bacillus subtilis* spores to establish its UVC lethal dose and determine the disinfection time required for various surfaces inside an ambulance. Similar studies in hospital settings using clinically relevant pathogens (*C. difficile*, MSRA, VRE, Norovirus, etc.) would be greatly beneficial. Correlations between UVC dose readings and a significant reduction in surface contamination would confirm the value of radiometry as a convenient surrogate for environmental cultures.

Our work has several limitations. First and foremost, due to limited time and available resources, we could not perform environmental cultures to validate our test modalities. Dose Verify™ cards have been internally validated for use by Clorox with their line of products, but the details are unknown to us. To our knowledge, we are the first third party to evaluate these cards. We could compare the results to radiometry readings, but not to cultures. Comparisons of card results, surface colony reduction and measured UVC dose would close the loop.

Our radiometer is a commercial hand-held unit with a flat sensor head that records incident light from a narrow angle. Further validation will be needed. Moreover, we have a small number of observations, for a limited number of surfaces in the patient room and bathroom. The energy estimation used in our live readings is probably an over-estimate, especially for the Enlight device, given the longer time required for the power output to plateau (we assume stable constant output for the entire cycle in our calculations).

## Conclusion

We used inexpensive methods (radiometry and commercial test cards) to test and compare UVGI devices. We did not find significant differences between the systems we tested, despite broadly different prices, marketing claims and workflows. Shadowing remains a problem for optimal irradiation, even with powerful devices and sensor systems.

There is clearly a need for standardisation of test methods, robust comparisons of UVGI systems, and more convenient ways to optimize UVGI devices in a specific environment. Objective criteria such as power output, bacterial count reduction, workflow and overall costs should be taken into account by the potential buyer. Although more external validation is needed, radiometry and test cards are potentially useful low-cost objective tools that could be adjuncts if not surrogates for environmental cultures for assessment, comparison and even continuous quality control of UVGI systems.

## Abbreviation

UVGI: Ultraviolet germicidal irradiation
